# Supplementary material for: Development and Validation of a Quick Sepsis-Related Organ Failure Assessment-Based Machine-Learning Model for Mortality Prediction in Patients with Suspected Infection in the Emergency Department
Source: J Clin Med. 2020 Mar 23;9(3):875. doi: 10.3390/jcm9030875 (PMC7141518; doi:10.3390/jcm9030875)
Supplement: Supplementary file 1 [file jcm-09-00875-s001.zip › supplementary 1.docx]

Supplementary appendix 1. The modified early warning score

| Score | 3 | 2 | 1 | 0 | 1 | 2 | 3 |
| --- | --- | --- | --- | --- | --- | --- | --- |
| Respiratory rate (beats/min) |  | ≤8 |  | 9–14 | 15–20 | 21–29 | >29 |
| Heart rate (beats/min) |  | ≤40 | 41–50 | 51–100 | 101–110 | 111–129 | >129 |
| Systolic blood pressure (mmHg) | ≤70 | 71–80 | 81–100 | 101–199 |  | ≥200 |  |
| Temperature (°C) |  | ≤35 | 35.1–36 | 36.1–38 | 38.1–38.5 | ≥38.6 |  |
| AVPU |  |  |  | Alert | Reacting to voice | Reacting to pain | Unresponsive |

AVPU: A for alert, V for reacting to vocal stimuli, P for reacting to pain, U for unconscious.
