# Supplementary material for: Development and Validation of a Quick Sepsis-Related Organ Failure Assessment-Based Machine-Learning Model for Mortality Prediction in Patients with Suspected Infection in the Emergency Department
Source: J Clin Med. 2020 Mar 23;9(3):875. doi: 10.3390/jcm9030875 (PMC7141518; doi:10.3390/jcm9030875)
Supplement: Supplementary file 1 [file jcm-09-00875-s001.zip › supplementary 3.docx]

Supplementary appendix 3. Predictive performance of algorithm-specific machine learning models through validation sets

| Algorithm | 3-day mortality | In-hospital mortality | 3-day ICU admission | ICU admission | qSOFA score ≥ 2 | qSOFA score < 2 |
| --- | --- | --- | --- | --- | --- | --- |
| BRF | 0.85 (0.79–0.86) | 0.78 (0.75–0.79) | 0.77 (0.75–0.78) | 0.75 (0.73–0.75) | 0.67 (0.57–0.75) | 0.84 (0.79–0.87) |
| XGB | 0.76 (0.74–0.79) | 0.76 (0.76–0.78) | 0.79 (0.79–0.79) | 0.78 (0.78–0.79) | 0.61 (0.58–0.65) | 0.79 (0.78–0.83) |
| LGBM | 0.70 (0.69–0.73) | 0.71 (0.69–0.73) | 0.78 (0.77–0.78) | 0.75 (0.74–0.75) | 0.60 (0.56–0.65) | 0.68 (0.64–0.71) |

BRF=balanced random forest; XGB=extreme gradient boosting; LGBM=light gradient boosting machine; ICU=intensive care unit; qSOFA=quick Sepsis-related Organ Failure Assessment score.
