# Supplementary material for: Development and Validation of a Quick Sepsis-Related Organ Failure Assessment-Based Machine-Learning Model for Mortality Prediction in Patients with Suspected Infection in the Emergency Department
Source: J Clin Med. 2020 Mar 23;9(3):875. doi: 10.3390/jcm9030875 (PMC7141518; doi:10.3390/jcm9030875)
Supplement: Supplementary file 1 [file jcm-09-00875-s001.zip › supplementary 4.docx]

Supplementary appendix 4. Algorithms of selected machine-learning models and results of training set cross-validation

| Model | Algorithm | The results of 5-fold cross validation  (AUROC, mean ± SD) |
| --- | --- | --- |
| 3-day mortality | BRF | 0.87 ± 0.02 |
| In-hospital mortality | BRF | 0.79 ± 0.01 |
| 3-day ICU admission | XGB | 0.83 ± 0.00 |
| ICU admission | XGB | 0.81 ± 0.01 |
| qSOFA score ≥ 2 | BRF | 0.66 ± 0.04 |
| qSOFA score < 2 | BRF | 0.78 ± 0.07 |

BRF=balanced random forest; ICU=intensive care unit; XGB=extreme gradient boosting; qSOFA=quick Sepsis-related Organ Failure Assessment score; AUROC=area under the receiver operating characteristics; SD= standard deviation.
